# DHCR24 Alleviates DNA Damage in Senescent Vascular Endothelial Cells via ENKUR/Ca^2+^ Signaling

**DOI:** 10.1111/acel.70688

**Published:** 2026-08-25

**Authors:** Han Li, Zhen Yang, Wukaiyang Liang, Jie Huang, Tianyi Ji, Hao Nie, Zixin Wan, Yuqi Qiu, Yi Huang, Le Zhang, Cuntai Zhang, Jinhua Yan

**Affiliations:** ^1^ Department of Geriatrics, Tongji Hospital, Tongji Medical College Huazhong University of Science and Technology Wuhan People's Republic of China; ^2^ Key Laboratory of Vascular Aging, Ministry of Education, Tongji Hospital of Tongji Medical College Huazhong University of Science and Technology Wuhan People's Republic of China

**Keywords:** 3β‐hydroxysterol δ 24 reductase, Ca^2+^ overload, DNA damage response, endoplasmic reticulum stress, mitochondrial dysfunction, vascular endothelial cell senescence

## Abstract

DNA damage is considered one of the major contributors to aging. DHCR24, a multifunctional enzyme located within the endoplasmic reticulum (ER), is closely related to DNA damage. Our previous study showed that DHCR24 could delay vascular endothelial cells (ECs) senescence. The relationship between DHCR24 and DNA damage during ECs senescence requires further investigation. Here, we demonstrate that aging activates ATM‐mediated DNA damage response (DDR) in human umbilical vein endothelial cells (HUVECs) and mouse pulmonary microvascular endothelial cells (PMVECs), and DHCR24 expression is downregulated. Knocking down DHCR24 in young HUVECs induces the activation of ATM‐mediated DDR, which has been confirmed in PMVECs of DHCR24 endothelial‐specific knockout mice. Consistently, RNAseq indicated that DHCR24 was essential for cell cycle regulation. Further investigations revealed that both replicatively senescent HUVECs and young HUVECs with DHCR24 knockout exhibited ER stress and mitochondrial dysfunction, which might be attributable to calcium overload resulting from DHCR24 deficiency. In this pathological process, the DHCR24‐deficiency‐induced upregulation of ENKUR markedly exacerbates calcium overload. Conversely, ENKUR knockdown not only alleviates the ER stress and mitochondrial dysfunction caused by DHCR24 inhibition, but also suppresses the ATM‐mediated DDR. Moreover, DHCR24 overexpression reduces the elevated ENKUR levels and simultaneously mitigates DOX‐induced calcium overload in HUVECs. Collectively, these findings identify DHCR24‐ENKUR‐dependent Ca^2+^ signaling as a mechanism linking ER‐mitochondrial homeostasis to endothelial DNA damage and senescence. Accordingly, restoring DHCR24 function or regulating calcium signal transduction through this pathway may hold therapeutic potential for delaying vascular ECs senescence and preventing age‐related diseases.

## Graphical abstract

In senescent vascular endothelial cells, DHCR24 deficiency triggers ENKUR‐dependent Ca^2+^ overload, which in turn induces endoplasmic reticulum stress and mitochondrial dysfunction, ultimately exacerbating oxidative stress and DNA damage.

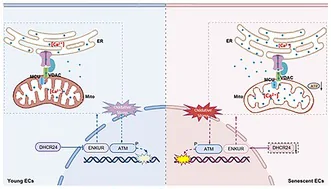

## 1 Introduction

Aging elevates cardiovascular disease risk in large part through age‐related endothelial dysfunction, which is the leading cause of chronic disability and mortality in the elderly (Suda et al. 2024, 2025; Xu et al. 2025). Vascular endothelial aging is the result of multiple mechanisms including oxidative stress, chronic inflammation, metabolic imbalance, and epigenetic disorders, leading to endothelial dysfunction (Bloom et al. 2023). Recent evidence suggests DNA damage as one important driver of aging‐related cellular dysfunction (Schumacher et al. 2021). Therefore, targeting endothelial DNA damage and its mechanistic role in aging provides a rationale for developing interventions against age‐related dysfunction and disease.

DHCR24, a multifunctional enzyme located within the endoplasmic reticulum (ER), is shown to confer protection against intracellular ER stress (Li et al. 2020). In our prior research, we demonstrated that DHCR24 inhibited reactive oxygen species (ROS) generation by activating the Caveolin‐1/ERK signaling axis, consequently delaying vascular ECs senescence and endothelial dysfunction (Li et al. 2024). ER stress and mitochondrial dysfunction are significant contributors to ROS overproduction (Wang et al. 2023). The ROS overproduction can lead to DNA damage response (DDR), which is characterized by the activation of ataxia telangiectasia mutated (ATM) to the site of damage and leading to Ser‐139 phosphorylation of histone H2A (γH2AX). γH2AX, adjacent to the site of DNA damage, also promotes the phosphorylated activation of transducer kinases Chk1 and Chk2, which converge signals on p53/p21 (Ren et al. 2026). Moreover, emerging evidence indicates that DHCR24‐mRNA slows the progression of doxorubicin (DOX)‐induced heart failure, which is closely linked to DNA damage (Zhang et al. 2025). These findings prompted us to systematically investigate the underlying relationship between DHCR24 and DNA damage in senescent vascular ECs.

Calcium serves as a critical intracellular second messenger that regulates a broad spectrum of molecular processes and cellular functions, such as cell proliferation, secretion, migration, and apoptosis (Giorgi et al. 2018). Accumulating evidence highlights calcium and calcium signaling as pivotal regulators of cellular senescence. In the neuroblastoma cell line SH‐SY5Y and human lung adenocarcinoma cell line A549, the intracellular calcium concentration was observed to increase in response to various aging stimuli (Triana‐Martínez et al. 2019; Yu et al. 2013). Furthermore, the application of calcium chelating agents effectively suppressed the DDR and the activation of the p53/p21 pathway (Yu et al. 2013). While the relationship between calcium signaling and DHCR24 during vascular ECs senescence remains to be elucidated. The increase in intracellular calcium is caused by both calcium influx and release from ER stores. Store‐operated Ca^2+^ entry (SOCE) activates when ER Ca^2+^ is depleted, allowing extracellular Ca^2+^ to enter and replenish the ER (Besprozvannaya et al. 2018). Calcium overload in the ER induces stress and excessive ROS production (Liu et al. 2023). Mitochondria are the key mediators of ER stress. ER stress‐induced Ca^2+^ release through mitochondrial‐associated membranes (MAMs), which enter the mitochondria via mitochondrial uniporter (MCU), thereby disrupting the electron transport chain (ETC), inducing mitochondrial membrane potential depolarization, dysfunction, and ultimately exacerbating the ROS production (Moreno et al. 2026). We hypothesize that DHCR24 may counteract endoplasmic reticulum stress and mitochondrial dysfunction via Ca^2+^ signaling.

The ENKUR (chromosome 10p12.1) was discovered via yeast two‐hybrid screening in TRPC channel studies, the mediators of Ca^2+^ cellular entry. Its encoded protein interacts with TRPC channels, acting as a regulator/effector (Sutton et al. 2004). Studies have shown that ENKUR suppresses lung adenocarcinoma progression by inhibiting PI3K/Akt and MAPK/ERK pathways, thereby reducing cell proliferation, migration, and invasion (Ma et al. 2019). Similarly, cinobufacin could induce ENKUR expression and activate β‐catenin/c‐Jun/MYH9 signaling, thereby reducing nasopharyngeal carcinoma metastasis (Hou et al. 2022). In human brain microvascular endothelial cells (HBMECs) treated with a cardiotonic steroid (Marinobufagenin, MBG), it was found that MBG enhanced the monolayer permeability of HBMECs and significantly increased the ENKUR expression (Ing et al. 2014). The ENKUR expression in megakaryocytes and platelets of myeloproliferative neoplasms is negatively correlated with the cell differentiation cycle gene CDC20, which may be related to the disorder of calcium homeostasis and ER stress (Seetharam et al. 2023). It is speculated that ENKUR may drive senescence by inducing proliferation/migration suppression and cell cycle arrest, which may be associated with intracellular calcium homeostasis.

Here, we have developed several vascular ECs senescence models to demonstrate DNA damage during vascular ECs senescence. Subsequently, we observed downregulated DHCR24 expression in DNA damage models. To investigate the direct link between DHCR24 and senescence‐associated DNA damage in endothelial cells, we constructed a DOX‐induced senescence model. Our results demonstrate that DHCR24 alleviates the DDR and associated cell cycle progression. Mechanistically, DHCR24 participates in the DDR by influencing ER homeostasis and mitochondrial function through regulation of the ENKUR‐mediated Ca^2+^ signaling pathway. These findings suggest that the DHCR24‐ENKUR‐Ca^2+^ axis is a mechanistic link between ER‐mitochondrial stress and endothelial senescence.

## 2 Materials and Methods

### 2.1 Cell Culture

Primary HUVECs were obtained from three different human umbilical cords, as previously described (Yan et al. 2017). The umbilical cords were collected with the donors' informed consent and approved by the Ethics Committee for Human Experiments of Tongji Hospital, Huazhong University of Science and Technology (TJ‐IRB20230419). Isolated HUVECs were inoculated in a culture flask (T25) and cultured to confluence in medium 199 supplemented with 10% fetal bovine serum (Biological Industries, Israel) and 2% low serum growth supplement (Gibco, USA). A replicative senescence model was established through the continuous passaging of young HUVECs until cell proliferation nearly ceased. In the experiments, young HUVECs were used at passage 5 (P5), while aged HUVECs were utilized at passage 13 (P13).

Primary pulmonary microvascular endothelial cells (PMVECs) were isolated as previously described (Sobczak et al. 2010). In summary, 100 μL sheep anti‐rat IgG Dynabeads (Thermo Fisher, USA) were mixed with 5 μg anti‐mouse PECAM‐1 antibody (BD, USA) and incubated overnight at 4°C. Freshly isolated mouse lung tissues were washed with pre‐cooled PBS, transferred to cryopreservation tubes, and dissected into small pieces. Subsequently, the lung tissue fragments were digested with type I collagenase under shaking conditions at 37°C for 45 min, passed through a sterile 70 μm nylon mesh filter, and then washed twice with 0.1% bovine serum albumin (BSA; Solaibao, China). To isolate the target cells, 30 μL PECAM‐1‐coated Dynabeads were added to the cell suspension and incubated for 25 min at room temperature. The cells were resuspended in DMEM supplemented with 20% FBS and seeded. When the cells reached 70%–80% confluence, a secondary purification step was performed using Dynabeads conjugated with anti‐ICAM‐2 antibody (BD, USA). Cells were cultured in a constant temperature incubator at 37°C with 5% CO_2_ atmosphere and subcultured with 0.05% trypsin–EDTA (Gibco, USA) at 80% confluence.

### 2.2 Cell Viability Assay

The cell viability was assessed using a cell counting kit‐8 (CCK‐8, Bimake, China), according to the manufacturer's instructions. Briefly, HUVECs were seeded into 96‐well plates. The cells were starved with 2% FBS for 12 h and then treated with DOX at different concentrations for 24, 48, and 72 h. Following treatment, 10 μL CCK‐8 solution was added to each well and incubated for 3 h at 37°C. The absorbance at 450 nm was measured by a microplate reader (Sunrise, Tecan, Switzerland).

### 2.3 Senescence‐Associated β‐Galactosidase (SA‐β‐Gal) Activity Assay

SA‐β‐gal activity was measured using Senescence β‐Galactosidase Staining Kit (Beyotime, China) and according to the manufacturer's protocol. Briefly, cells were seeded into 12‐well plates. After the necessary stimulation, the cells were fixed in a fixative solution at room temperature for 15 min and then incubated in the new staining solution at 37°C without CO_2_ overnight. The cells were observed and visualized under an inverted ordinary light microscope (OLYMPUS CKX41). Blue staining cells and a total number of cells were counted using Image J.

### 2.4 Cell Proliferation Assay

Edu incorporation assay was examined using an iClickTM Edu Andy Flour 555 Imaging Kit (Wuhan ABP‐Biosciences Co. Ltd. China), according to the manufacturer's instructions. Briefly, cells seeded in 12‐well plates were cultured in a 2% FBS starvation medium for 12 h. After pretreatment with 0.05 μM DOX for 48 h and incubation with normal culture medium for 3 days, the cells were treated with 10 μM Edu for another 2 h. Next, the cells were fixed with 3.7% formaldehyde PBS for 15 min and treated with 0.5% Triton X‐100 permeable membrane for 20 min. The cells were subsequently incubated with an iClick reaction liquid for 30 min at room temperature away from light. The cells were then counterstained with 5 μg/mL Hoechst 33342 and observed under an inverted fluorescence microscope (OLYMPUS IX71). Image J was used to count five random fields to determine the number of Edu‐positive and DAPI‐positive nuclei.

### 2.5 RNA Extraction and Quantitative RT‐PCR (q‐PCR)

Total RNA was isolated and purified using the RNA purification kit (Magen, China), as suggested by the manufacturer. An equal amount of total cellular RNAs was reversely transcribed into cDNA using a ReverTra Ace qPCR RT kit (TOYOBO, Japan). The qPCR reaction mixture contained 5 μL SYBR Green Realtime PCR Master Mix (TOYOBO, Japan), 2.5 μL DNase‐free water, 1.5 μL cDNA (5 ng/μl), and 0.5 μL of each forward and reverse primer. Amplification and quantification of qPCR were performed with the Step One Plus system (Applied Biosystems, USA) using the following protocol: 60 s at 95°C, 40 cycles of 15 s at 95°C, 15 s at the annealing temperature (60°C), extension for another 45 s at 72°C, and another 15 s at 95°C, 60 s at 60°C, and 15 s at 95°C for the melt curve stage. The GAPDH was used as the internal reference gene, and the 2^−∆∆Ct^ method was used to calculate relative mRNA expression. The sequence of primers was synthesized by the Sangon Biotech Co. Ltd. (Shanghai, China), listed in Table 1.

**TABLE 1:** The primer sequences used in this study.

Gene	Forward primer (5′–3′)	Reverse primer (3′–5′)
GAPDH	TCCAAAATCAAGTGGGGCGA	AAATGAGCCCCAGCCTTCTC
DHCR24	ATGCACTCCGTCCGAAAACT	CGAAACGCAGCTTGACGTA
E2F1	CATCCCAGGAGGTCACTTCTG	GACAACAGCGGTTCTTGCTC
MCM2	ATGGCGGAATCATCGGAATCC	GGTGAGGGCATCAGTACGC
ORC1	TGGATCGAAAACTGCACTACC	CTGGATGTGAATCTCGGTGGA
ORC6	ACAAGGAGACATATCAGAGCTGT	AGTGGCCTGGATAAGTCAAGAT
CDC6	CCAGGCACAGGCTACAATCAG	AACAGGTTACGGTTTGGACATT
CDC20	GCACAGTTCGCGTTCGAGA	CTGGATTTGCCAGGAGTTCGG
CDC25	TCTACGGAACTCTTCTCATCCAC	TCCAGGAGCAGGTTTAACATTTT
CDC45	TTCGTGTCCGATTTCCGCAAA	TGGAACCAGCGTATATTGCAC
CCNA2	GGATGGTAGTTTTGAGTCACCAC	CACGAGGATAGCTCTCATACTGT
CCNB1	AATAAGGCGAAGATCAACATGGC	TTTGTTACCAATGTCCCCAAGAG
CDK1	AAACTACAGGTCAAGTGGTAGCC	TCCTGCATAAGCACATCCTGA
PLK1	AAAGAGATCCCGGAGGTCCTA	GGCTGCGGTGAATGGATATTTC
MCU	ACCGGACGGTACACCAGAG	GATAGGCTTGAGTGTGAACTGAC
VDAC	ACGTATGCCGATCTTGGCAAA	TCAGGCCGTACTCAGTCCATC
ENKUR	TTCCAGTCCCTCTCGGTCTTTATAG	TTCAATTATGCCAATGTCGTGTTCTAG

### 2.6 Western Blot

RIPA lysis buffer (Boster, China) containing protease and phosphatase inhibitors was used to prepare total proteins. Protein concentrations were measured using BCA Protein Assay Kit (Boster, China). Equal amounts of proteins were separated by 8%–12% sodium dodecyl sulfate‐polyacrylamide gel electrophoresis (SDS‐PAGE) before being transferred to polypropylene difluoride membranes (Millipore, USA). The membranes were blocked with 5% non‐fat milk at room temperature for 1 h and incubated with primary antibodies at 4°C overnight. Antibodies for human DHCR24 (#2033S, 1:1000, Cell Signaling Technology, USA), SIRT1 (#8469S, 1:1000, Cell Signaling Technology, USA), P16 (Affinity‐#AF5484, Proteintech‐#10883‐1‐AP, 1:500, China), p‐P53 (#9284, 1:1000, Cell Signaling Technology, USA), P53 (#2524, 1:1000, Cell Signaling Technology, USA), p‐ATM (ab81292, 1:1000, Abcam, UK), γH2AX (#9718S, 1:1000, Cell Signaling Technology, USA), CDK1 (#ab133327, 1:1000, Abcam, UK), CyclinA2 (#ab181591, 1:1000, Abcam, UK), CyclinB1 (#ab32053, 1:1000, Abcam, UK), p‐IRE1 (#AP1442, 1:1000, ABclonal, China), IRE1 (#A21021, 1:1000, ABclonal, China), VDAC (#4866, 1:1000, Cell Signaling Technology, USA), OPA1 (#A28496, 1:1000, ABclonal, China) and MCU (#14997, 1:1000, Cell Signaling Technology, USA) were used. In addition, antibodies for GAPDH (#10494‐1‐AP, 1:4000, Proteintech, China) were used as a protein loading control. The membranes were then incubated with horseradish peroxidase (HRP)‐conjugated secondary antibodies (1:5000; Promotor, China) for 1 h. Eventually, the membranes were processed with an enhanced chemiluminescence kit (Beyotime, China). Band density measurement was performed using Image J software. For analysis, band densities were normalized to internal reference, also in terms of the gray value of the target protein divided by the gray value of GAPDH.

### 2.7 Cell Cycle Analysis

The cells were grown in Petri dishes with a diameter of 6 cm. For cell cycle analysis, cells were digested with 0.05% trypsin and collected in flow cytometry tubes. The cells were fixed with 70% ethanol and sheltered from light at 4°C overnight. The cells were washed with PBS and incubated with 0.5 mL PI/RNase staining buffer solution (BD Pharmingen, USA) at room temperature for 30 min. Cell cycle progression was assessed using FACScan Flow Cytometer (BD Biosciences, USA), and data were analyzed using Flow Jo 10.6 software (BD Biosciences, USA).

### 2.8 Immunofluorescence Analysis

The cells were seeded on the cover glass. After treatment, the cells were washed thrice with PBS and fixed for 15 min with 4% paraformaldehyde at room temperature. After washing, cell membranes were permeabilized with 0.5% Triton‐100X and blocked with 5% goat serum at room temperature for 1 h. They were then incubated with mouse anti‐8‐OHdG primary antibody (#sc‐66036, 1:100, Santa Cruz, USA) overnight at 4°C, followed by fluorescent secondary antibody (#AS057, 1: 500, ABclonal, China) for 1 h. Anti‐fluorescence quenching tablets containing DAPI (Boster, China) were used to seal sections and identify nuclei.

The en face immunofluorescence staining was consistent with the methods described in previous literature (Wang et al. 2019). Mouse aortas pre‐perfused with 4% paraformaldehyde were isolated and subsequently longitudinally incised along the vascular wall. Aortas were permeabilized for 10 min in PBS containing 0.1% Triton X‐100, blocked with 10% normal goat serum in TBS (supplemented with 2.5% polysorbate 20) for 1 h at room temperature then incubated with rabbit anti‐γH2AX (#9718S, 1:100, Cell Signaling Technology, USA) overnight at 4°C, followed by secondary antibody (#AS058, 1:500, ABclonal, China) for 1 h at room temperature. Anti‐fluorescence quenching tablets containing DAPI (Boster, China) were used to seal sections and identify nuclei.

The cells and aortas were visualized and photographed using a Nikon C2 confocal microscope.

### 2.9 siRNA Interference

The sequence of siRNA targeting DHCR24 is 5′‐GCTGAATAGCATTGGCAATT‐3′, the ENKUR sequence is 5′‐ACACGACATTGGCATAATT‐3′, and the negative control siRNA sequence is 5′‐TTCTCCGAACGTGTCACGTdTdT‐3′. They were designed and synthesized by RiboBio (RiboBio, China). When HUVECs density reached 50%–70%, siRNAs (30 nM) transfection was conducted for 48 h using lipo3000 transfection reagent (Thermo Fisher, USA) according to the manufacturer's protocol. In particular, Lipofectamine 3000 was performed using a starvation medium containing 2% fetal bovine serum and replaced 6 h later with a complete medium containing 10% fetal bovine serum. Gene silencing efficiency was verified by q‐PCR.

### 2.10 RNA‐Sequencing Analysis

To identify differentially expressed genes between two different samples, transcript abundance was quantified using RSEM (https://deweylab.github.io/RSEM/) and normalized as transcripts per million (TPM). DESeq2 was used for differential expression analysis. Foldchange ≥ 1 and *p* value < 0.01 are regarded as differential gene expression thresholds. In addition, functional enrichment analyses including GO and KEGG were performed with bonferroni corrected *p* values ≤ 0.05 were used to identify differentially expressed genes that were significantly enriched. GO enrichment was performed using Goatools (https://github.com/tanghaibao/Goatools), and KEGG pathway enrichment was performed using KOBAS (http://kobas.cbi.pku.edu.cn/home.do). The RNA‐seq data have been submitted to Sequence Read Archive (https://www.ncbi.nlm.nih.gov/sra/, BioProject ID: PRJNA943440).

### 2.11 Lentiviral Overexpression of DHCR24

The lentivirus for DHCR24 overexpression was named OE‐DHCR24, and the control lentivirus was named CON. Cells were transfected with the lentiviral production purchased from GeneChem (Shanghai, China) according to the manufacturer's protocol. The vector name is “GV492” and the element order was “UB‐MCS‐3FLAG‐CBH‐gcGFP‐IREs‐puromycin”. Briefly, 500 μL mixture consisting of virus diluent (MOI = 100), HitansG, and complete culture medium was evenly mixed and allowed to stand for 10 min before being added to HUVECs inoculated in 12‐well plates. Notably, 8 h after transfection, the complete culture medium should be replaced, and 0.5 μg/mL puromycin could be used for screening at 72 h. Gene overexpression efficiency was verified by immunoblotting.

### 2.12 Animals

Vascular endothelium‐specific DHCR24 knockout mice were constructed and validated as described previously (Li et al. 2024). Mice carrying the floxed *DHCR24* allele were crossed with *Tie2*‐Cre^+^ mice to generate endothelium‐specific *DHCR24*‐knockout mice. Male C57BL/6 mice, both young (3 months old) and elderly (14 months old) were purchased from Vital River Laboratories. All mice were kept in a specific pathogen‐free (SPF) grade sterile environment at a constant room temperature (22°C ± 2°C), humidity (40%–60%) and a 12:12‐h light/dark cycle. They were provided with a standard mouse diet and ad libitum access to water. The experimental procedures and animal care protocols were approved by the Laboratory Animal Welfare & Ethics Committee of Tongji Hospital, Huazhong University of Science and Technology (TJH‐202108010).

### 2.13 Calcium Content Analysis

Fluo4‐AM was used for Ca^2+^ measurement. For all Ca^2+^ assays, cells were seeded at the same density in 6‐well plates before experiments to exclude the effect of cell density. 1 million cells were harvested and resuspended in 250 μL hanks solution. Subsequently, 250 μL medium containing Fluo4‐AM was added, resulting in a final Fluo4‐AM concentration of 2 μM. The mixture was incubated at 37°C for 30 min in the dark. After washing once with hanks solution, the cells were resuspended in 500 μL hanks solution for flow cytometry analysis.

### 2.14 Transmission Electron Microscopy (TEM)

Vascular tissues were collected and fixed in 1.25% glutaraldehyde/0.1 M phosphate buffer (PH 7.4) for 2 h at room temperature. After rinsing thrice, the tissues were successively dehydrated in 50%–70%–80%–90%–95%–100%–100% alcohol for 15 min each time. Acetone: 812 embedding agent = 1:1 mixture infiltration overnight, pure 812 embedding agent infiltration overnight. Polymerization at 60°C for 48 h. Uranium and lead double staining performed after sectioning. Mitochondrial ultrastructure was observed by transmission electron microscopy (Japan).

### 2.15 ATP Content Detection

The ATP assay kit (Beyotime, China) was used following the manufacturer's protocol to measure the ATP content in HUVECs. The cells were lysed and centrifuged at 12,000 *g* for 5 min at 4°C, and the supernatant was used for the assay. A standard curve created using ATP standards was used to measure the amount of ATP in the supernatants. By comparing luminescence readings to the standard curve, ATP content was calculated. Protein concentrations were determined using BCA Protein Assay Kit (Boster, China). To eliminate errors due to differences in protein amounts, ATP concentrations were converted to nmol/mg protein.

### 2.16 Mitochondrial Membrane Potential Detection

Mitochondrial membrane potential was measured using a JC‐1 probe (HY‐15534, MCE, China). The treated cells were seeded in confocal dishes and cultured overnight. After washing thrice with HBSS (containing Ca^2+^/Mg^2+^), the cells were incubated with JC‐1 working solution (5 μg/mL) at 37°C in the dark for 30 min. Then, they were washed thrice and visualized and photographed under the confocal microscope.

### 2.17 Mitochondrial ROS Production

Mitochondrial ROS were examined using MitoSOX Red (HY‐D1055, MCE, China). After seeding treated cells in confocal dishes and culturing overnight, the sample was washed thrice with HBSS (containing Ca^2+^/Mg^2+^) and then incubated with MitoSOX Red working solution (2.5 μg/mL) and Hoechst 33342 (5 μg/mL) at 37°C for 20 min in the dark. Following another three washes, the cells were visualized and photographed under the confocal microscope.

### 2.18 Statistical Analysis

All statistical analyses were performed using IBM SPSS software (version 22.0). The data were presented as the means ± standard deviation (SD) and analyzed by Student's *t*‐test, one‐way ANOVA or two‐way ANOVA. Each experiment was independently repeated at least three times (*N* ≥ 3). Statistical differences were determined to be significant at *p* values < 0.05.

## 3 Results

### 3.1 DHCR24 Was Down‐Regulated in ATM‐Mediated DNA Damage Response

**FIGURE 1:**
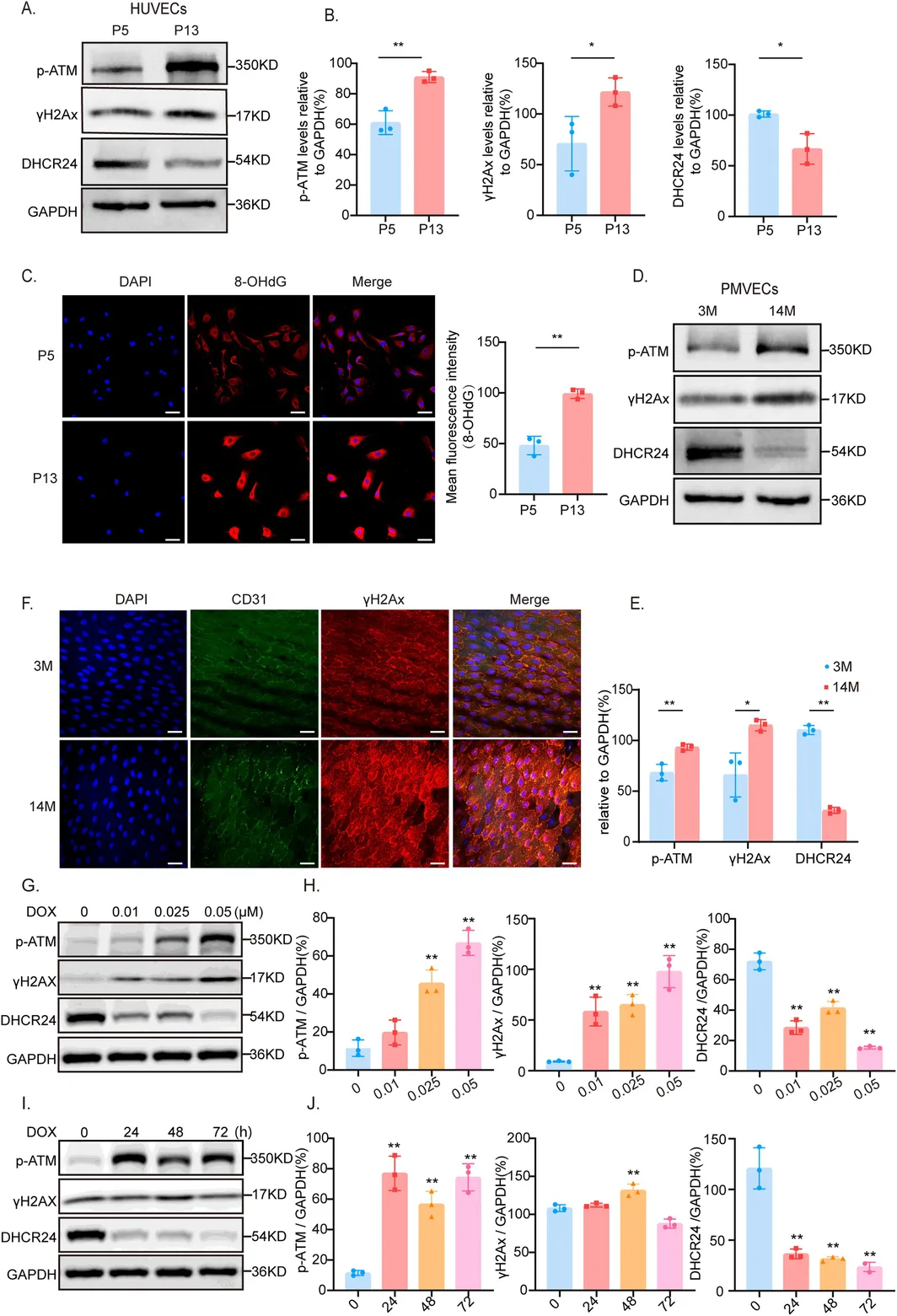
DHCR24 was down‐regulated in ATM‐mediated DNA damage response. (A, B) DHCR24, p‐ATM, and γH2Ax expression in young (P5) vs. old (P13) HUVECs. (C) Immunofluorescent staining for 8‐OHdG (red) and nuclei (blue) in young (P5) vs. old (P13) HUVECs. Scale bar, 50 μm. (D, E) Western blot analysis of p‐ATM, and γH2Ax expression in the PMVECs from young (3 months) and aged (14 months) mice. (F) Expression of γH2Ax (red) and CD31 (green) in aortas from young (3 months) and aged (14 months) mice. Scale bar, 25 μm. (G) DHCR24, γH2Ax and p‐ATM were detected using western blotting in HUVECs that had been treated with a dose‐dependent DOX for 48 h, followed by cultured in normal medium for three days. (H) Quantitative data of the protein levels. (I) DHCR24, γH2Ax and p‐ATM were detected using western blotting in HUVECs that had been treated with time‐dependent DOX (0.05 μM), followed by cultured in normal medium for three days. (J) Quantitative data of the protein levels. *n* ≥ 3. The values are presented as mean ± SD. *p < 0.05, **p < 0.01.

DNA damage accumulation is one of the key factors that trigger cellular senescence. To investigate DNA damage during ECs senescence, we performed studies in replicative senescent HUVECs and naturally aged mice. As shown in Figure 1A–C, significant DNA damage was observed in senescent HUVECs by western blot analysis of γH2Ax expression and 8‐OHdG staining. Then, we isolated PMVECs from young (3‐month‐old) and aged (14‐month‐old) mice to further assess the age‐related accumulation of DNA damage. We employed multiple markers to confirm the senescent state: p16 expression and the percentage of SA‐β‐gal staining increased significantly in PMVECs isolated from aged mice, compared to that from young mice, whereas SIRT1 expression and cell proliferation capacity decreased (Figure S1A–D). Most importantly, the level of γH2Ax increased during aging (Figure 1D,E). Consistent with this expression pattern, there was a marked increase of γH2Ax level in aorta tissues of aged mice compared to that of the young mice, as shown by immunofluorescence (Figure 1F).

Our previous studies have shown that silencing DHCR24 increases the intracellular reactive oxygen species (ROS) level, while ATM activation is involved in oxidative stress‐induced endothelial dysfunction and premature senescence (Li et al. 2024; Zhan et al. 2010). Therefore, we examined p‐ATM levels in senescent ECs and found that ATM was activated upon the initiation of DNA damage (Figure 1A,B,D,E). These results suggest ECs senescence activates ATM‐mediated DNA damage response.

To directly link DHCR24 to DNA damage in senescent cells, we employed a doxorubicin (DOX)‐induced DNA damage senescent model in HUVECs. First, the expression levels of SIRT1, p16, SA‐β‐gal activity assay, and cell proliferation assay were used to assess cellular senescence. HUVECs were treated with 0, 0.01, 0.025, and 0.05 μM DOX for 48 h, followed by a 3‐day incubation in normal medium. SIRT1 was down‐regulated, and p16 was up‐regulated after DOX treatment for 48 h at different concentrations, all in a dose‐dependent manner (Figure S2A,B). Then, HUVECs were treated with 0.05 μM DOX for 0, 24, 48, and 72 h, followed by a 3‐day incubation in normal medium. While treated with 0.05 μM DOX for different times, the changes were presented at 48 h (Figure S2C,D). Combined with the effect of cell viability (Figure S2E,F), the concentration of 0.05 μM and induction time of 48 h were applied in subsequent experiments requiring DOX treatment. The results show that, compared with the control group, the percentage of blue‐staining cells in DOX‐induced group was significantly increased (Figure S2G,H), and the Edu‐positive rate was significantly decreased (Figure S2I,J). The results above suggested that the premature senescence model was successfully established. Most importantly, they demonstrate that DOX‐induced senescence in HUVECs triggered ATM‐mediated DNA damage responses and concurrently downregulated DHCR24 expression (Figure 1G–J).

### 3.2 DHCR24 Knockdown Induces ATM‐Mediated DNA Damage Response

**FIGURE 2:**
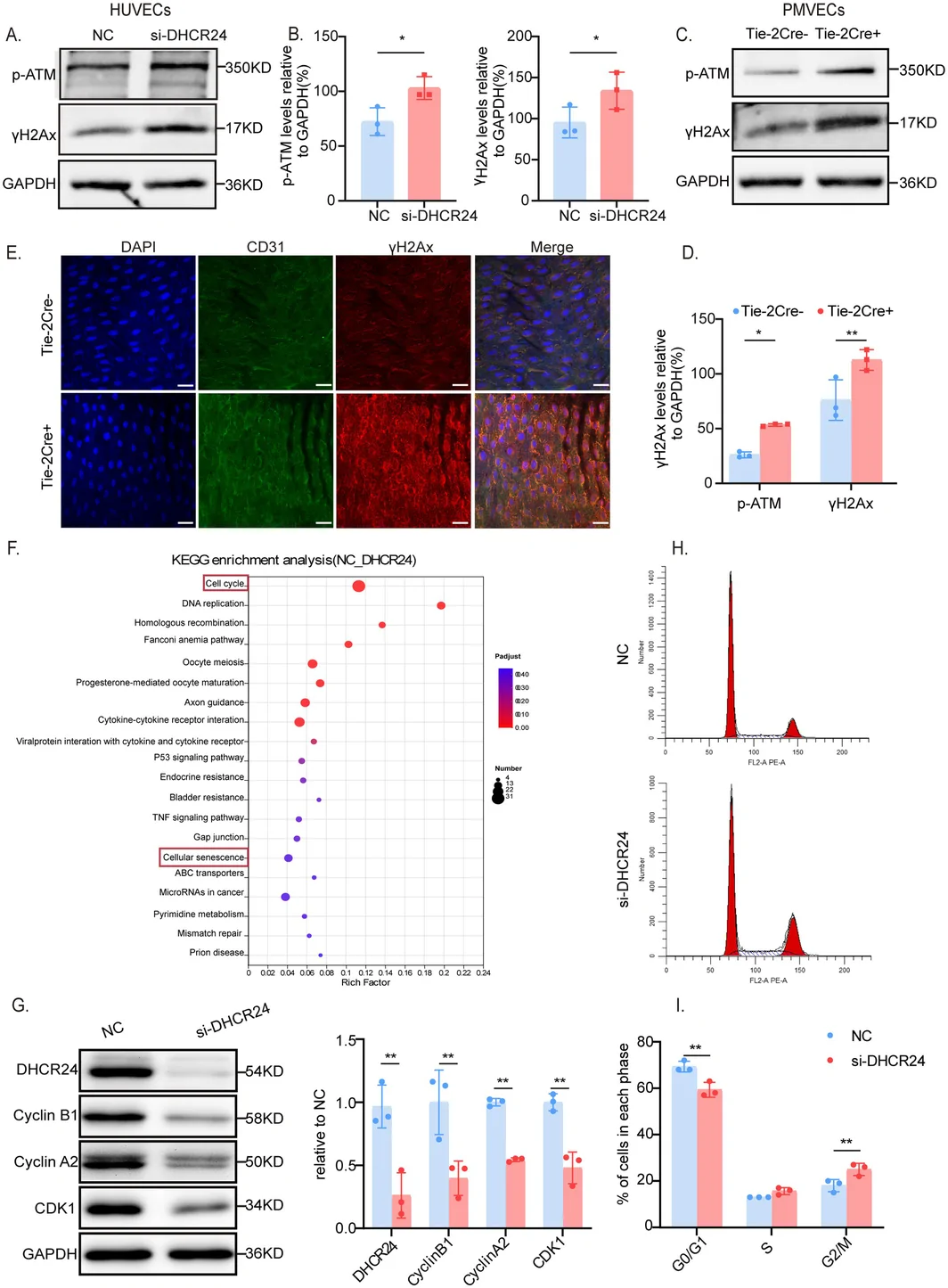
DHCR24 knockdown induces ATM‐mediated DNA damage response. (A, B) p‐ATM, and γH2Ax expression in young HUVECs (P5) treated with siRNA targeting the human DHCR24. (C, D) p‐ATM, and γH2Ax expression PMVECs from *DHCR24* ^*flox/flox*^ *‐Tie2Cre* ^*−*^ mice and *DHCR24* ^*flox/flox*^ *‐Tie2Cre* ^+^ mice. (E) Expression of γH2Ax (red) and CD31 (green) in aortas from *DHCR24* ^*flox/flox*^ *‐Tie2Cre* ^*−*^ mice and *DHCR24* ^*flox/flox*^ *‐Tie2Cre* ^+^ mice. Scale bar, 25 μm. (F) KEGG enrichment analysis. (G) CDK1, CyclinA2, CyclinB1 were detected using western blotting after DHCR24 silencing and quantitative data of the protein levels. (H, I) DHCR24 silencing arrests cell cycle in G2 phase. Representative images of flow cytometry analysis (H) and cell cycle proportion quantification (I). *n* ≥ 3. The values are presented as mean ± SD. *p < 0.05, **p < 0.01.

To elucidate the role of DHCR24 in the DNA damage response, we knocked down DHCR24 using siRNA in young HUVECs (P5). DHCR24 knockdown cells showed a significant increase in γH2AX and p‐ATM compared with NC group (Figure 2A,B). To further investigate the role of DHCR24 in DNA damage response, we employed *DHCR24* ^*flox/flox*^ *‐Tie2Cre* ^+^ mice and their littermate controls. Our results demonstrated that the SA‐β‐gal staining positive rate in PMVECs from *DHCR24* ^*flox/flox*^ *‐Tie2Cre* ^+^ mice was significantly elevated, while the tubule formation was markedly reduced (Figure S3A–D). Consistently, the PMVECs from *DHCR24* ^*flox/flox*^ *‐Tie2Cre* ^+^ mice showed increased γH2AX and p‐ATM levels (Figure 2C,D). Immunofluorescence staining of the aortas from *DHCR24* ^*flox/flox*^ *‐Tie2Cre* ^+^ mice also showed a significant increase in γH2AX (Figure 2E). The results indicate that DHCR24 silencing exacerbates the ATM‐mediated DNA damage responses.

To better understand DHCR24 function, RNA‐seq and KEGG analysis were used to investigate the transcriptome of negative control (“NC”) and DHCR24 silencing (“si‐DHCR24”) cells. KEGG enrichment analysis showed that the biological function of DHCR24 was tightly correlated with cell cycle regulation (Figure 2F). Cluster analysis was performed for DEGs associated with cell cycle and senescence and it was found that CDK1, CyclinA2, CyclinB1, etc. were significantly downregulated (Figure S3E). To validate the results of RNA‐seq, the cycle‐related DEGs were measured at the mRNA and found that CDK1, cyclinA2, and cyclinB1 were down‐regulated (Figure S3F). Cyclin B1 and CyclinA2 interact with CDK1 protein kinase to form a serine/threonine kinase holoenzyme complex, is critical for controlling the cell cycle at the G2/M transition (Henglein et al. 1994). The protein levels were consistent with the gene expression results (Figure 2G). Meanwhile, flow cytometry analysis showed that after DHCR24 silencing, compared with negative control, the proportion of G1 and G2 phase cells decreased and increased, respectively (Figure 2H,I). DNA damage response directly induces cell cycle arrest, and these findings indirectly corroborate the role of DHCR24 in regulating the DNA damage response.

### 3.3 DHCR24 Overexpression Alleviates ECs DNA Damage and Senescence

**FIGURE 3:**
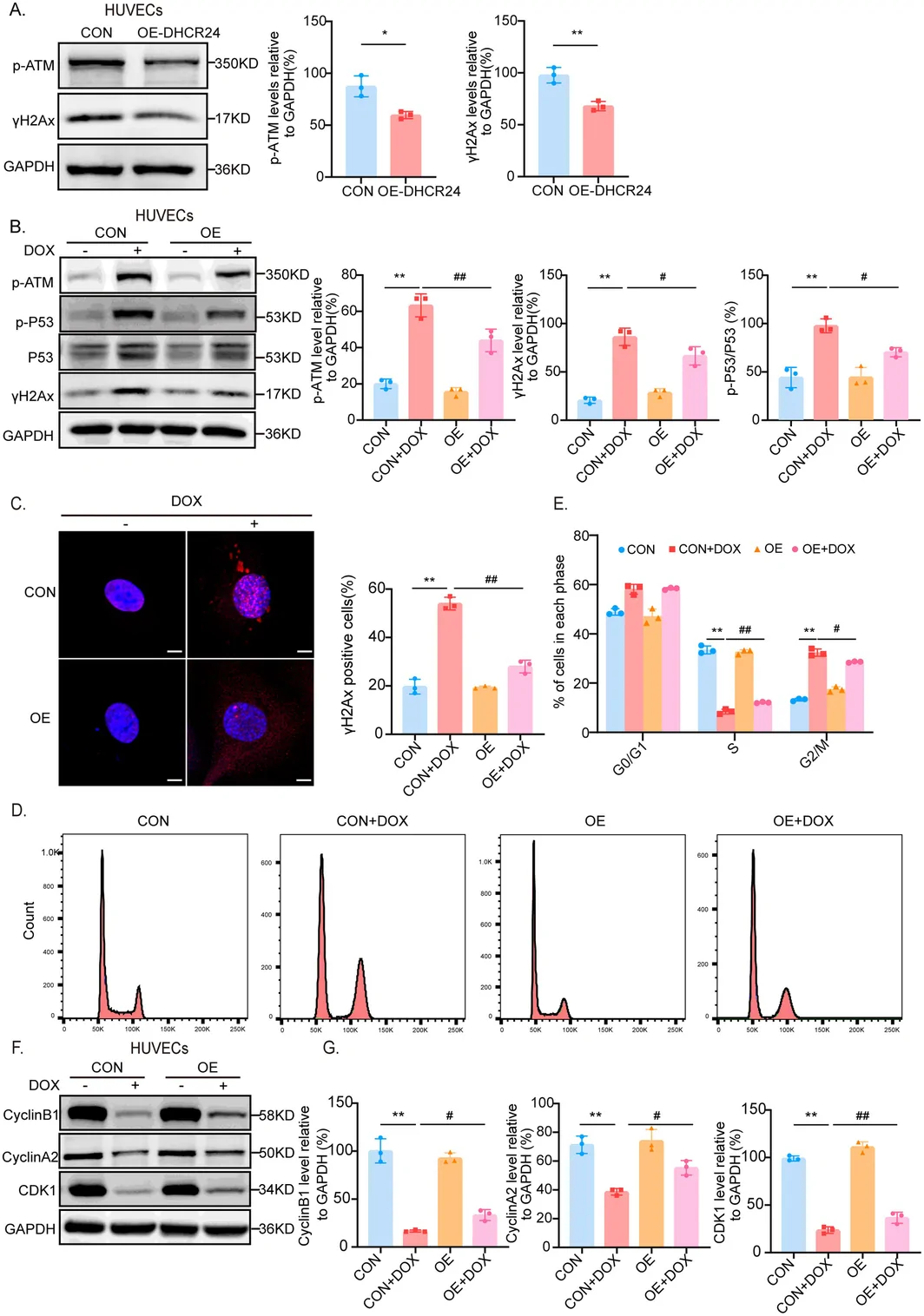
DHCR24 overexpression alleviates ECs DNA damage and senescence. (A) p‐ATM, and γH2Ax expression in old HUVECs (P13) treated with DHCR24 lentivirus. (B) γH2AX, p‐P53, P53, and p‐ATM were detected after DHCR24 overexpression in DOX treated HUVECs, followed by a 3‐day incubation in normal medium, and quantitative data. (C) The immunofluorescence staining of γH2AX foci was performed. Nuclei were counterstained with DAPI. Scale bar, 25 μm. Cells with no less than 20 foci were scored as γH2AX‐positive cells. (D, E) DHCR24 overexpression improved DOX‐induced cell cycle arrest. Representative images of flow cytometry analysis (D) and cell cycle proportion quantification (E). (F, G) CDK1, CyclinA2, CyclinB1 were detected using western blotting after DHCR24 overexpression in DOX treated HUVECs, followed by a 3‐day incubation in normal medium, and quantitative data. *n* ≥ 3. The values are presented as mean ± SD. *p < 0.05, **p < 0.01, compared to CON group without DOX. ^#^ *p* < 0.05, ^##^ *p* < 0.01, compared to CON group with DOX.

To evaluate whether DHCR24 mitigates DNA damage during vascular ECs senescence, we overexpressed DHCR24 via lentiviral transduction. Intriguingly, DHCR24 overexpression in senescent HUVECs (P13) significantly reduced the γH2AX and p‐ATM levels compared with the CON group (Figure 3A).

Moreover, DHCR24 overexpression attenuated DOX‐induced premature senescence in HUVECs, as demonstrated by senescence‐associated proteins, SA‐β‐gal staining, cell proliferation (Figure S4A–F). Consistently, DHCR24 overexpression also reduced the excessive intracellular ROS production in HUVECs upon DOX stimulation (Figure S4G,H). To investigate the role of DHCR24 in suppressing ATM‐mediated DDR, we analyzed the DNA damage marker γH2AX and key components of the DDR pathway. DHCR24 overexpression reduced the high level of γH2AX after DOX treatment (Figure 3B), which was consistent with the immunofluorescence results (Figure 3C). DDR activates the p53 pathway after ATM activation (Ren et al. 2026), so the phosphorylation levels of p53 and ATM were detected. Similarly, the phosphorylation levels of p53 and ATM proteins were significantly increased after DOX induction, which was reversed by DHCR24 overexpression (Figure 3B). Since DOX induces DNA damage (Zhuang et al. 2020), which triggers cell cycle arrest, cell cycle distribution was examined after DOX induction. Flow cytometry analysis revealed that after DOX treatment, the proportion of G2 phase cells increased significantly compared with the CON group, and DHCR24 overexpression could alleviate DOX‐induced G2 phase stagnation (Figure 3D,E). Similarly, western blot analysis displayed that DOX treatment decreased levels of CDK1, CyclinA2 and CyclinB1, which was reversed by DHCR24 overexpression (Figure 3F,G). These findings reveal that DHCR24 reduces DNA damage and antagonizes ATM activation, which collectively mitigates ECs senescence.

### 3.4 DHCR24 Deficiency Promotes ER Stress and Mitochondrial Impairment in Senescent ECs

**FIGURE 4:**
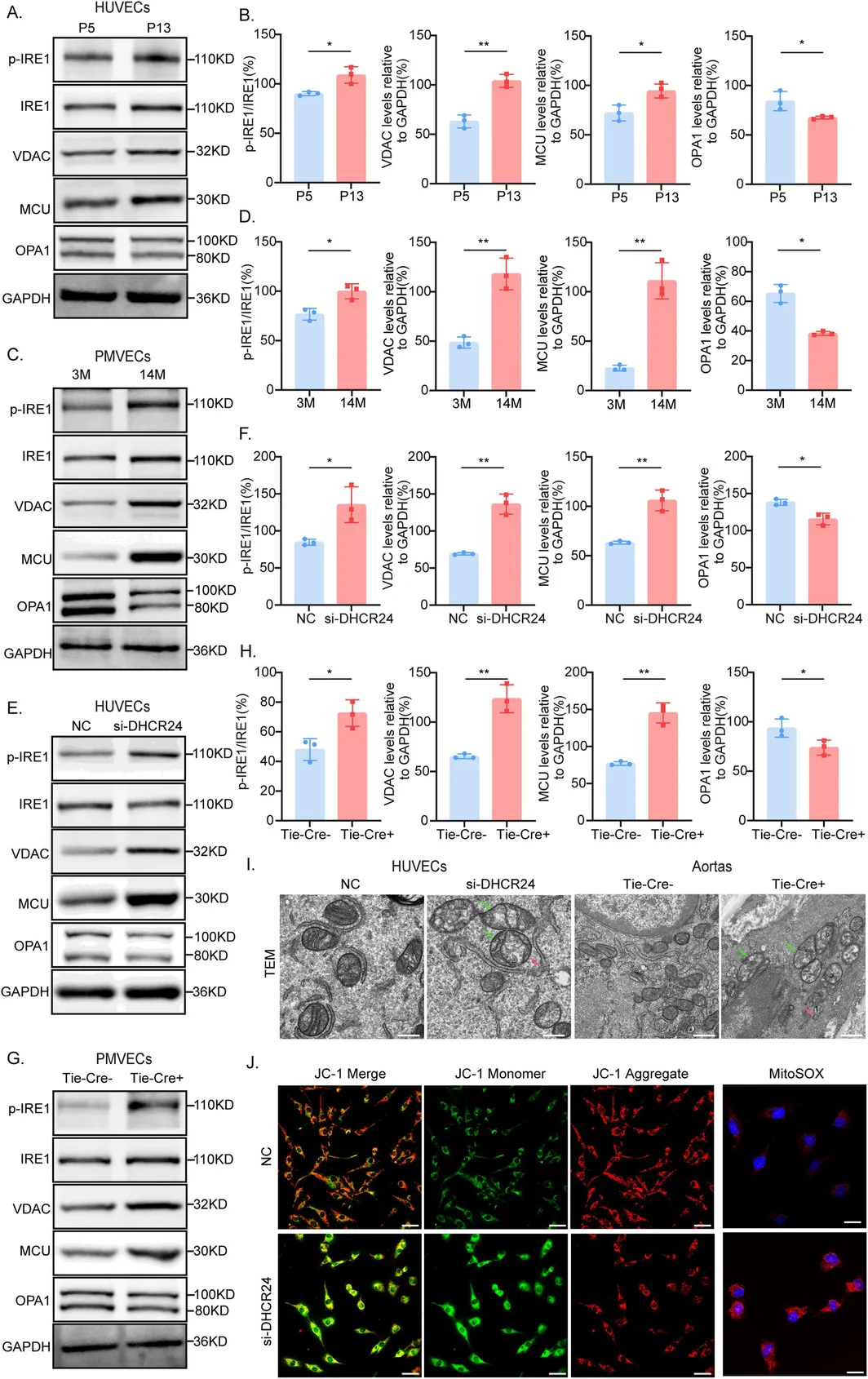
DHCR24 deficiency promotes ER stress and mitochondrial impairment in senescent ECs. (A, B) Western blot analysis in young (P5) vs. old (P13) HUVECs. (C, D) Western blot analysis in the PMVECs from young (3 months) and aged (14 months) mice. (E, F) Western blot analysis in young HUVECs (P5) treated with siRNA targeting the human DHCR24. (G, H) Western blot analysis in the PMVECs from *DHCR24* ^*flox/flox*^ *‐Tie2Cre* ^*−*^ mice and *DHCR24* ^*flox/flox*^ *‐Tie2Cre* ^+^ mice. (I) Representative TEM micrographs show the ultrastructural details of the ER and mitochondria in young HUVECs (P5) after siRNA targeting human DHCR24 treatment and aortas from *DHCR24* ^*flox/flox*^ *‐Tie2Cre* ^*−*^ mice and *DHCR2* ^*flox/flox*^ *‐Tie2Cre* ^+^ mice. Scale bar, 500 nm. (J) Mitochondrial membrane potential evaluated by JC‐1 (Scale bar, 50 μm) and mitochondrial ROS generation evaluated by MitoSox Red (Scale bar, 25 μm). *n* ≥ 3. The values are presented as mean ± SD. *p < 0.05, **p < 0.01.

Oxidative stress induced by ER stress is one of the major sources of DNA damage, and mitochondrial dysfunction serves as a critical mediator in ER stress‐induced DNA damage (Moreno et al. 2026). Given our previous finding that DHCR24 inhibits ROS excessive production (Li et al. 2024), we further examined its role in ER stress and mitochondrial dysfunction during ECs senescence. In senescent HUVECs and PMVECs, western blotting demonstrated elevated p‐IRE1/IRE1 levels, increased VDAC and MCU expression, and reduced OPA1 expression, suggesting that ER stress and mitochondrial dysfunction are concurrently induced during endothelial senescence (Figure 4A–D). Consistent with the findings in senescent HUVECs and PMVECs, DHCR24‐knockdown cells and PMVECs from *DHCR24* ^*flox/flox*^ *‐Tie2Cre* ^+^ mice displayed a similar molecular signature (Figure 4E–H). Moreover, DHCR24‐knockdown HUVECs displayed overt mitochondrial damage (shrinkage, swelling, and cristae rupture) as well as vesicle dilatation and sparseness of ER, as compared with control cells. Consistently, in the thoracic aortas from *DHCR24* ^*flox/flox*^ *‐Tie2Cre* ^+^ mice, similar ultrastructural abnormalities were observed, including marked mitochondrial swelling, cristae rupture, and ER degeneration (Figure 4I). We further measured mitochondrial membrane potential and oxidative stress markers. DHCR24‐knockdown cells exhibited increased JC‐1 monomer/green fluorescence and reduced aggregate/red fluorescence (indicative of ΔΨm loss), along with elevated MitoSOX Red fluorescence (reflecting mitochondrial ROS overproduction) (Figure 4J) (Figure S5A,B). These results suggest that DHCR24 deficiency drives both structural damage and functional impairment of ER and mitochondria. Mechanistically, DHCR24 may be involved in regulating ER stress and mitochondrial dysfunction during ECs senescence.

### 3.5 DHCR24 Regulates Calcium Overload in Senescent ECs

**FIGURE 5:**
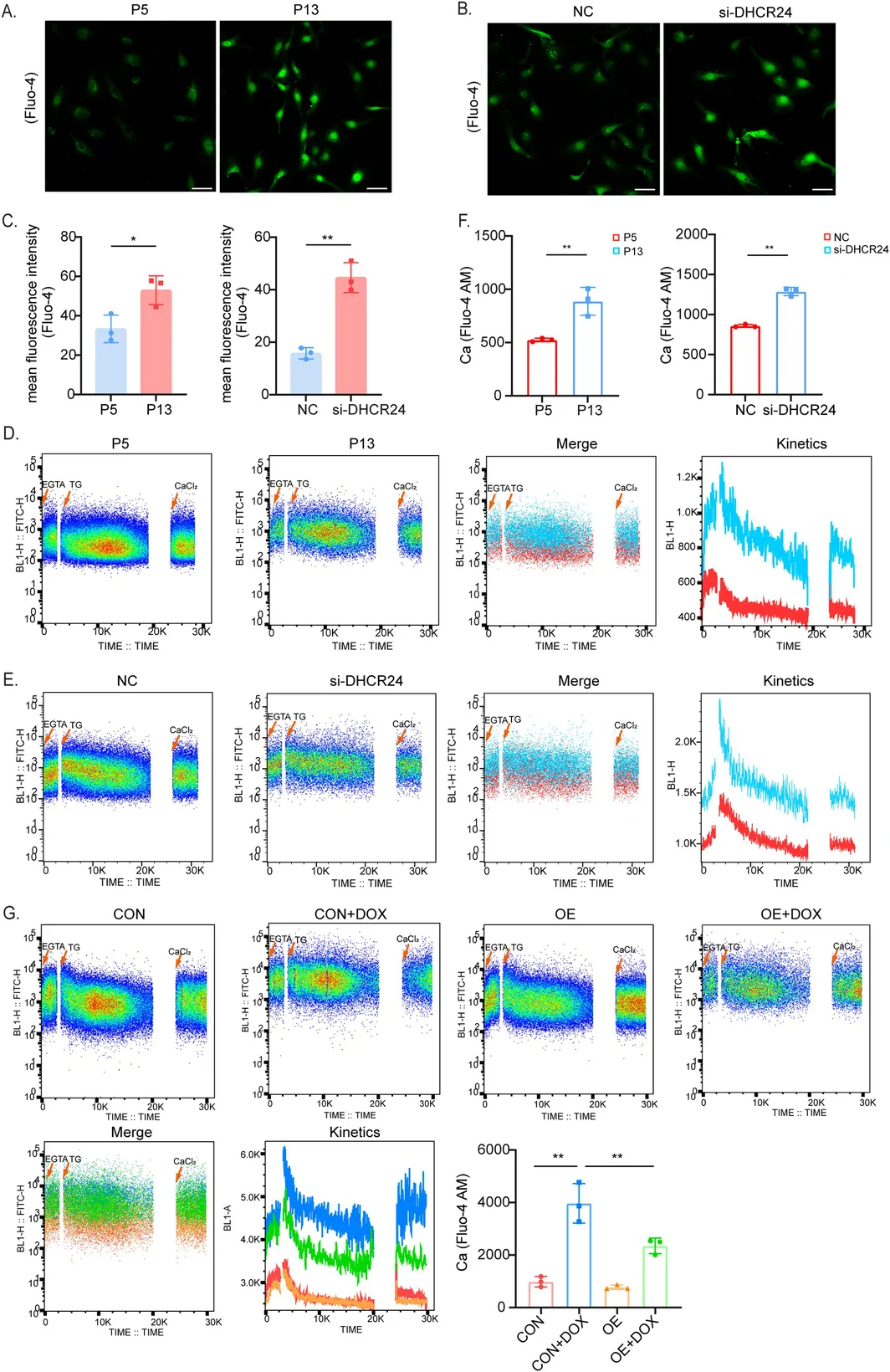
DHCR24 regulates calcium overload in senescent ECs. (A, B) Representative fluorescence imaging of intracellular Ca^2+^ Scale bar, 50 μm. (C) Quantitative data of mean fluorescence intensity (Fluo4) in senescent HUVECs and DHCR24 knockdown‐induced HUVECs. (D–F) ER Ca^2+^ concentrations were detected in senescent HUVECs and DHCR24 knockdown‐induced HUVECs. (G) ER Ca^2+^ concentrations were detected after DHCR24 overexpression in DOX treated HUVECs, followed by a 3‐day incubation in normal medium, and quantitative data. *n* ≥ 3. The values are presented as mean ± SD. *p < 0.05, **p < 0.01.

Intracellular calcium overload serves as a critical mechanism underlying ER stress and mitochondrial dysfunction, resulting in excessive ROS production and cellular senescence. We assessed cellular Ca^2+^ using Fluo4‐AM fluorescent probes. The results indicated that the intracellular Ca^2+^ concentration was significantly increased both in replicative senescent HUVECs (P13) and in DHCR24 knockdown‐induced HUVECs (si‐DHCR24) (Figure 5A–C). To separate thapsigargin‐releasable Ca^2+^ (mostly from ER, hereafter referred to as ER Ca^2+^) and Ca^2+^ influx from outside of cells (SOCE), Ca^2+^ in hanks solution was chelated by EGTA before thapsigargin treatment and then 2 mM Ca^2+^ was added back to induce Ca^2+^ influx. We found ER Ca^2+^ concentrations were increased in senescent HUVECs and DHCR24 knockdown‐induced HUVECs (Figure 5D–F). Consistently, in DOX‐induced prematurely senescent HUVECs, DHCR24 overexpression significantly attenuated the DOX‐evoked elevation of ER Ca^2+^ levels (Figure 5G). To examine SOCE in senescent cells, we examined the intracellular Ca^2+^ concentration after thapsigargin treatment and observed that SOCE was activated in senescent HUVECs and DHCR24 knockdown‐induced HUVECs (Figure S5C–F). Collectively, these results support that DHCR24 may help preserve ER Ca^2+^ homeostasis during endothelial senescence, thereby limiting calcium overload‐associated ER stress and mitochondrial dysfunction.

### 3.6 ENKUR Mediates Endothelial Calcium Overload Induced by DHCR24 Deficiency

**FIGURE 6:**
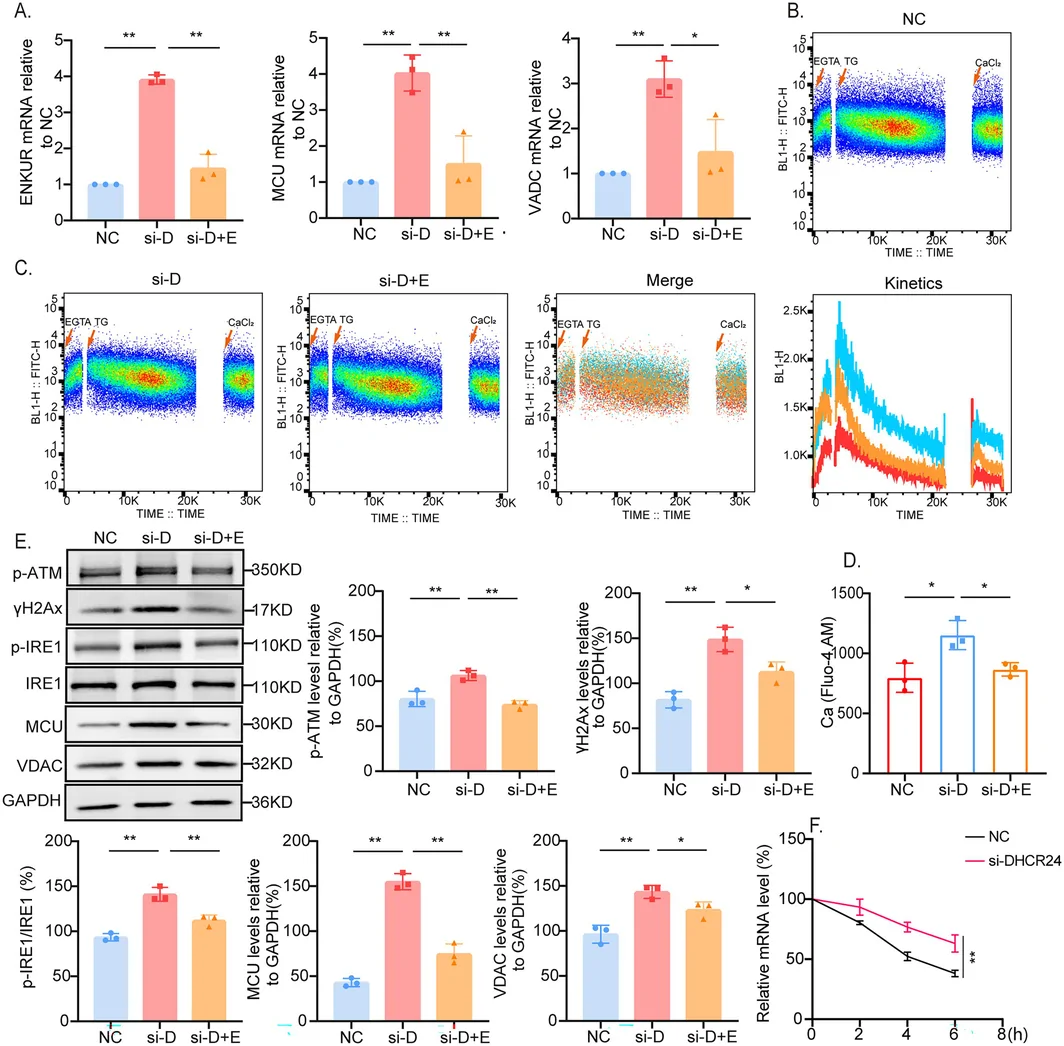
ENKUR mediates endothelial calcium overload induced by DHCR24 *deficiency*. (A) q‐PCR analysis in young HUVECs (P5) treated with siRNA targeting the human DHCR24 and ENKUR. (B–D) ER Ca^2+^ concentrations were detected in DHCR24 and ENKUR double knockdown HUVECs. (E) Western blot analysis of p‐ATM, γH2Ax, p‐IRE1, IRE1, VDAC and MCU expression in young HUVECs (P5) treated with siRNA targeting the human DHCR24 and ENKUR. (F) Actinomycin D chase assay showing ENKUR mRNA stability up to 6 h after Actinomycin D treatment (5 μg/mL). *n* ≥ 3. The values are presented as mean ± SD. *p < 0.05, **p < 0.01.

ENKUR can function as a Ca^2+^ sensor. RNA‐seq results showed that 384 genes were up‐regulated and ENKUR exhibited significant up‐regulation (Figure S6A), which were further validated by qPCR (Figure S6B). To explore the role of DHCR24 in regulating ENKUR, we extended the siRNA knockdown study to double knock‐downs of DHCR24 and ENKUR. Compared with the DHCR24 knockdown group, qPCR analysis revealed a downregulation of ENKUR expression in the double knockdown group, consistent with the changes in MCU and VDAC expression (Figure 6A). Compared with DHCR24 knockdown group, the intracellular Ca^2+^ level was decreased in DHCR24 and ENKUR double knockdown group (Figure 6B–D). Consistently, western blot analysis demonstrated that the protein levels of p‐ATM, γH2AX, p‐IRE1, VDAC and MCU were significantly reduced in the double knockdown group relative to the DHCR24 knockdown group (Figure 6E). Additionally, intracellular ATP levels supported these results (Figure S6C). Moreover, knockdown of ENKUR also significantly reversed intracellular Ca^2+^ overload compared with senescent HUVECs (Figure S6D,E). To investigate the mechanism by which DHCR24 regulates ENKUR expression, we performed actinomycin D chase assays to characterize the degradation kinetics of ENKUR mRNA upon transcriptional inhibition. Following blockade of de novo RNA synthesis by actinomycin D, the degradation rate of ENKUR mRNA was markedly lower in the DHCR24 knockdown group relative to the control group (Figure 6F). And DHCR24 overexpression also reduced the high expression of ENKUR mRNA in the DOX‐induced HUVECs senescence (Figure S6F). These results indicate that DHCR24 alleviates Ca^2+^overload, ER stress and mitochondrial dysfunction by promoting ENKUR mRNA degradation, thereby weakening the ATM‐mediated DNA damage response.

## 4 Discussion

The present study suggests a potential axis through which DHCR24 may contribute to vascular endothelial homeostasis, possibly involving ENKUR‐mediated calcium signaling. Our findings extend the known functions of DHCR24 beyond its established role in cholesterol biosynthesis, and implicate ENKUR as an important factor that links calcium dynamics to cellular senescence and genomic instability.

In our prior research, we demonstrated that DHCR24 delays ECs senescence and endothelial dysfunction by regulating cholesterol metabolism and scavenging excessive intracellular ROS. Studies have demonstrated that ROS induce ECs senescence and endothelial dysfunction through multiple mechanisms, including transcription inhibition, inflammatory signaling activation, and organelle and macromolecular damage, contributing to cardiovascular disease progression (Qi et al. 2024; Saito‐Takatsuji et al. 2021; Yan et al. 2024). Since DNA contains information about all the proteins and RNAs produced by cells, DNA damage is a potent inducer of cellular senescence, via activation of ataxic telangiectasia mutated serine/threonine kinase (ATM) and Rad 3‐associated serine/threonine kinase (ATR), which then activates p53 and p21, leading to cell cycle arrest and cellular senescence (Barnes et al. 2022). Therefore, identifying and intervening in the upstream mechanisms of DNA damage represents a core strategy for delaying ECs senescence and preventing associated diseases.

This study elucidated the activation of the ATM‐mediated DDR during vascular ECs senescence. When replicative senescence occurs, telomeres shorten to critical lengths, causing exposed chromosome ends to be recognized as DNA damage. This activates the DDR, where DNA damage is a secondary result of telomere loss (Adam et al. 2025). Increased cell division leads to mitochondrial dysfunction and excessive ROS production, which in turn promotes DNA damage accumulation and accelerates the aging process (Ahmed and Lingner 2018; De Lange et al. 2007). To investigate the role of DHCR24 in DNA damage as an upstream trigger of senescence, DOX‐induced DNA damage senescent model in HUVECs was established. The results showed that DHCR24 overexpression suppressed DOX‐induced ATM‐dependent DNA damage, alleviating cell cycle arrest, and mitigated premature senescence. Additionally, DHCR24 was identified as a regulator of ATM‐mediated DDR in the endothelial replicative senescence model. Transcriptome analysis further substantiated the role of DHCR24 in cell cycle regulation. Based on our previous research findings, DHCR24 may alleviate DNA damage by inhibiting ROS. However, further studies are needed to elucidate the potential mechanisms for preventing ROS‐dependent DNA damage.

Given the critical role of ROS in inducing DNA damage (Shadfar et al. 2023), the mechanism by which DHCR24 participates in ROS regulation was investigated subsequently. The gradient of intracellular and extracellular calcium concentrations is essential for sustaining cellular normal function. A reduction in the transmembrane calcium concentration gradient, resulting from elevated intracellular calcium levels, is regarded as one of the critical indicators of cell senescence (Berchtold and Villalobo 2025). Ca^2+^ is a ubiquitous signaling molecule capable of controlling a variety of cellular processes. Ca^2+^ signaling is initiated through the release of Ca^2+^ from intracellular organelles or via influx from the extracellular environment. ER is not only the synthesis site of proteins and lipids, but also the main intracellular Ca^2+^ storage and signal transduction center (Groenendyk and Michalak 2023). Depletion of ER Ca^2+^ using the ER Ca^2+^‐ATPase (SERCA) inhibitor thapsigargin (TG) is known to cause ER dysfunction and activate the unfolded protein response (UPR) (Pontisso et al. 2024). Recent studies have found that ER Ca^2+^ overload activates IRE1, induces ER stress, and increases ROS production (Liu et al. 2023; Zhao et al. 2023; Zhu et al. 2018). In the present study, IRE1 activation was observed in senescent HUVECs and PMVECs, with an increase in cellular Ca^2+^ concentration, particularly in ER, as well as enhanced store‐operated Ca^2+^ entry (SOCE). Similarly, the same phenomenon was observed in premature senescent ECs induced by DHCR24 deficiency, suggesting that DHCR24 may protect against ROS‐mediated DNA damage and ECs senescence by regulating ER Ca^2+^ homeostasis.

The ER is a key component of the cellular reticular network (CRN), which includes the Golgi apparatus, lysosomes, peroxisomes, endocytic pathway components, and the nuclear envelope. The ER establishes contact sites with the plasma membrane and mitochondria for communication (Groenendyk and Michalak 2023). ER‐mitochondrial contact sites, also known as mitochondria‐associated membranes (MAMs), elucidate how mitochondrial Ca^2+^ accumulation depends on the ER (Wang et al. 2025, 2024). Ca^2+^ can be released from the ER through the inositol 1,4,5‐trisphosphate receptor (IP3R) and the voltage‐dependent anion channel (VDAC1) on the mitochondrial outer membrane. These channels are interconnected by GRP75 and localized at the MAM (Xian et al. 2024). In addition, MCU is the major active transporter of Ca^2+^ through the inner mitochondrial membrane to the mitochondrial matrix, and mitochondrial functions including ATP synthesis, mitochondrial membrane potential and tricarboxylic acid cycle are all affected by the influx of Ca^2+^ into the mitochondrial matrix (Wang et al. 2025; Xian et al. 2024). Excessive calcium influx into mitochondria can affect mitochondrial homeostasis and induce oxidative stress, increasing ROS levels (Wang et al. 2024). In this study, we found that VDAC, MCU expression was significantly up‐regulated during both replicative senescence and DHCR24 deficiency‐induced senescence in HUVECs. Mitochondrial swelling, cristae fragmentation, vacuolization and ER vesicle dilatation degeneration were observed in mouse aortic endothelium and in young HUVECs with DHCR24 knockdown, suggesting that ER calcium overload induced by DHCR24 deficiency may be involved in ER stress and mitochondrial dysfunction. This may be an important mechanism by which DHCR24 protects endothelial cells from oxidation‐dependent DNA damage.

Studies have shown that DOX can induce intracellular Ca^2+^ disorder, elevates ER stress‐related protein levels, and disrupts mitochondrial membrane potential in rat ventricular myocytes (Chen et al. 2017). Our study also confirmed that DOX‐treated HUVECs showed increased intracellular calcium levels, whereas DHCR24 overexpression reversed this effect. This finding was consistent with the results for intracellular ROS levels. A limitation of this study is that the effect of DHCR24 overexpression on the ER and mitochondrial functions in DOX‐induced endothelial cells was not directly validated. However, in light of the current findings, DHCR24‐mediated regulation of calcium homeostasis may be critical for maintaining ER and mitochondrial function.

To further explore the molecular mechanism of DHCR24 regulating ER calcium overload, we further analyzed the differentially expressed genes in the transcriptome and found that ENKUR was significantly up‐regulated in the DHCR24 deletion group. Further validation revealed that knocking down ENKUR alleviated the Ca^2+^ homeostasis imbalance, ER stress, and mitochondrial dysfunction induced by DHCR24 deficiency, thereby reducing DNA damage and the activation of ATM. ENKUR encodes a protein (Enkurin) that interacts with calmodulin and several transient receptor potential canonical (TRPC) cation channel proteins, acting as adaptors to localize the signal transduction machinery to calcium channels and mediate Ca^2+^ influx (van Vliet and Agostinis 2017). The study by Seetharam SM et al. demonstrated that ER Ca^2+^ depletion downregulates enkurin expression, which promotes cell proliferation (Seetharam et al. 2023). Hou et al. demonstrated that ENKUR inhibits β‐catenin/c‐Jun/MYH 9 signaling, thereby reducing UBE 3A‐mediated p53 ubiquitination and degradation (Hou et al. 2022). This is consistent with our results and together supports that high ENKUR expression accelerates cellular senescence and is related to intracellular Ca^2+^ homeostasis. Another limitation of this study is that all mechanistic experiments were performed in endothelial models, including HUVECs, PMVECs, and endothelial‐specific DHCR24‐deficient mice. Therefore, the present findings do not determine whether the DHCR24‐ENKUR/Ca^2+^ axis is endothelial cell‐specific or also operates in other senescent cell types, such as IMR90 fibroblasts. Future studies using non‐endothelial senescence models will be necessary to evaluate the broader applicability of this mechanism.

In conclusion, this study suggests that DHCR24 maintains ER and mitochondrial function, protects against endothelial cell senescence, and reduces DNA damage through the regulation of ENKUR‐dependent Ca^2+^ homeostasis, thereby offering a new perspective for the prevention of DNA damage and senescence.

## Author Contributions

Jinhua Yan and Cuntai Zhang conceived the study and designed the experiments. Han Li, Zhen Yang, and Wukaiyang Liang performed the experiments. Jie Huang and Tianyi Ji analyzed the data, Han Li, Zixin Wan, Yuqi Qiu, and Hao Nie prepared the figures. Yi Huang and Le Zhang provided experimental technical support. Han Li, Zhen Yang Wrote the first version of the manuscript, Jinhua Yan made logical and content revisions to the manuscript. All authors approved the final version of the manuscript.

## Funding

This work was supported by the Joint Funds of the National Natural Science Foundation of China (grant number U24A20741), and the National Natural Science Foundation of China Youth Science Fund Project (grant numbers 82301754 and 82501876).

## Conflicts of Interest

The authors declare no conflicts of interest.

## Supporting information


**Figure S1:** The senescent phenotype of PMVECs in naturally aging mice.
**Figure S2:** DOX‐induced HUVECs senescence.
**Figure S3:** The senescence phenotype of PMVECs in DHCR24 endothelial‐specific knockout mice and the RNA‐seq analysis and verification.
**Figure S4:** DHCR24 overexpression alleviates DOX‐induced HUVECs senescence.
**Figure S5:** Mitochondrial function analysis and calcium overload in senescent HUVECs.
**Figure S6:** ENKUR knockdown alleviated calcium overload and mitochondrial dysfunction.

## Data Availability

The data that supports the findings of this study are available in the Supporting Material of this article.
