# Supplementary material for: DHCR24 Alleviates DNA Damage in Senescent Vascular Endothelial Cells via ENKUR/Ca2+ Signaling
Source: Aging Cell. 2026 Aug 25;25(9):e70688. doi: 10.1111/acel.70688 (PMC13504672; doi:10.1111/acel.70688)
Supplement: Supplementary file 1 [file ACEL-25-e70688-s001.docx]

## Supplementary materials

Figure S1. The senescent phenotype of PMVECs in naturally aging mice.

Figure S2. DOX-induced HUVECs senescence.

Figure S3. The senescence phenotype of PMVECs in DHCR24 endothelial-specific knockout mice and the RNA-seq analysis and verification.

Figure S4. DHCR24 overexpression alleviates DOX-induced HUVECs senescence.

Figure S5. Mitochondrial function analysis and calcium overload in senescent HUVECs.

Figure S6. ENKUR knockdown alleviated calcium overload and mitochondrial dysfunction.

**Materials and methods**

**2.1	Tube Formation Assay**

To assess the angiogenic capacity of PMVECs, a Matrigel-based tube formation assay was performed. Matrigel (Corning, USA) was thawed overnight at 4 °C and uniformly coated into pre-chilled 96-well plates (50 μl/well). Following this, the plates were transferred to a 37 °C, 5% CO₂ incubator for 40 min. Cells were then seeded into Matrigel-coated plates at a density of 1×10^4^ cells per well and incubated for 6 h. Vascular networks were visualized and imaged under an inverted light microscope. Quantitative analysis of tube formation parameters—including the number of nodes and total branch length—were conducted using the "Angiogenesis Analyzer" plugin in Image J software.

**2.2	Intracellular ROS detection**

The intracellular ROS were detected using Dihydroethidium (HY-D0079, MCE, China). After seeding treated cells in confocal dishes and culturing overnight, the sample was washed thrice with HBSS (containing Ca²⁺/Mg²⁺) and then incubated with Dihydroethidium working solution (10 μM) at 37 °C for 20 min in the dark. Following another three washes, the cells were visualized and photographed under the confocal microscope.

**Figure S1.**

**Figure S1. The senescent phenotype of PMVECs in naturally aging mice.**

(A-B). Western blot analysis of SIRT1 and p16 expression in the PMVECs from young (3 months) and aged (14 months) mice. (C). SA-β-gal staining in PMVECs from young (3 months) and aged (14 months) mice. Scale bar, 100 μm. (D). Edu for the cell proliferation of PMVECs from young (3 months) and aged (14 months) mice. Scale bar, 50 μm. n ≥ 3. The values are presented as mean ± SD. *^*^P < 0.05, ^**^P < 0.01*.

**Figure S2.**

**Figure S2. DOX-induced HUVECs senescence.**

(A). Senescence-associated proteins were detected using western blotting in HUVECs that had been treated with a dose-dependent DOX for 48 h, followed by cultured in normal medium for three days. (B). Quantitative data of the protein levels. (C). Senescence-associated proteins were detected using western blotting in HUVECs that had been treated with time-dependent DOX (0.05 μM), followed by cultured in normal medium for three days. (D). Quantitative data of the protein levels. (E). HUVECs were treated with the indicated doses of DOX for 48 h. (F). HUVECs were treated with DOX (0.05 μM) for 0, 24, 48, and 72 h. (G-H). SA-β-gal staining in HUVECs that had been treated with 0.05 μM DOX for 48 h, followed by cultured in normal medium for three days. Scale bar, 25 μm. (I-J). EdU assay in HUVECs that had been treated with 0.05 μM DOX for 48 h, followed by cultured in normal medium for three days. Scale bar, 50 μm. n ≥ 3. The values are presented as mean ± SD. **P < 0.05, **P < 0.01*, compared to the control group without DOX.

**Figure S3.**

**Figure S3. The senescence phenotype of PMVECs in DHCR24 endothelial-specific knockout mice and the RNA-seq analysis and verification.**

(A-B). Representative images and quantification of SA-β-gal staining in PMVECs from DHCR24*^flox/flox^*-Tie2Cre^-^ mice and DHCR24*^flox/flox^*-Tie2Cre^+^ mice. Scale bar, 50 μm. (C-D). Representative images and quantification of tube formation were shown for PMVECs from DHCR24*^flox/flox^*-Tie2Cre^-^ mice and DHCR24*^flox/flox^*-Tie2Cre^+^ mice. Scale bar, 100 μm. (E). Cluster analysis of cell cycle-related genes. (F). q-PCR analysis of cycle-related genes expression in HUVECs after DHCR24 silencing. n ≥ 3. The values are presented as mean ± SD. **P < 0.05, **P < 0.01.*

**Figure S4.**

**Figure S4. DHCR24** **overexpression alleviates DOX-induced HUVECs senescence.**

(A). Senescence-associated proteins were detected using western blotting after DHCR24 overexpression in DOX-treated HUVECs, followed by a 3-day incubation in normal medium. (B). Quantitative data of the protein levels. (C-D). SA-β-gal assay was performed after DHCR24 overexpression in DOX treated HUVECs, followed by a 3-day incubation in normal medium. Senescent cells were observed (C), and the percentages of SA-β-gal positive cells (D). Scale bar, 25 μm. (E-F). EdU assay was performed after DHCR24 overexpression in DOX treated HUVECs, followed by a 3-day incubation in normal medium. The representative images (E) and the percentages of cells that incorporated Edu into DNA (F). Scale bar, 50 μm. (G). Intracellular ROS levels were measured in DOX-treated HUVECs following DHCR24 overexpression and a subsequent 3-day incubation in normal medium. Scale bar, 50 μm. (H). Mean fluorescence Intensity of ROS. n ≥ 3. The values are presented as mean ± SD. **P* < 0.05, ***P* < 0.01, compared to CON group without DOX. *#P* < 0.05, *##P* < 0.01, compared to CON group with DOX.

**Figure S5.**

**Figure S5. Mitochondrial function analysis and calcium overload in senescent HUVECs.**

(A). The ratio of fluorescence intensity of JC-1 aggregate to that of JC-1 monomer. (B). Mean fluorescence intensity of MitoSox. (C, D). Representative image of Fluo4 fluorescence intensity over time and quantitative data of mean fluorescence intensity in senescent HUVECs. (E, F). Representative image of Fluo4 fluorescence intensity over time and quantitative data of mean fluorescence intensity in young HUVECs after DHCR24 silencing. n ≥ 3. The values are presented as mean ± SD. **P < 0.05, **P < 0.01.*

**Figure S6.**

**Figure S6. ENKUR Knockdown alleviated calcium overload and mitochondrial dysfunction.**

(A). Volcano plots of differentially expressed gene and FPKM value of ENKUR in “NC” and “si-DHCR24” group. (B). q-PCR analysis of DHCR24 and ENKUR in young HUVECs (P5) treated with siRNA targeting the human DHCR24. (C). The ATP content in young HUVECs (P5) treated with siRNA targeting the human DHCR24 and ENKUR. (D-E). Representative image of Fluo4 fluorescence intensity over time and quantitative data of mean fluorescence intensity in young HUVECs (P5) and old HUVECs (P13) treated with siRNA targeting ENKUR*.* (F). ENKUR levels assay after DHCR24 overexpression in DOX treated HUVECs，followed by a 3-day incubation in normal medium. n ≥ 3. The values are presented as mean ± SD. **P < 0.05, **P < 0.01.*

.
